# Comparison of unprocessed silk cocoon and silk cocoon middle layer membranes for guided bone regeneration

**DOI:** 10.1186/s40902-016-0057-1

**Published:** 2016-02-29

**Authors:** Seong-Gon Kim, Min-Keun Kim, HaeYong Kweon, You-Young Jo, Kwang-Gill Lee, Jeong Keun Lee

**Affiliations:** 1 Department of Oral and Maxillofacial Surgery, College of Dentistry, Gangneung-Wonju National University, Gangneung, Gangwondo 210-702 Republic of Korea; 2 Sericultural and Apicultural Materials Division, National Academy of Agricultural Science, Suwon, South Korea; 3 Department of Oral and Maxillofacial Surgery, Ajou University School of Medicine, Suwon, South Korea

**Keywords:** Guided bone regeneration, Silk cocoon, Membrane, Bone defect

## Abstract

**Background:**

Silk cocoon is composed of multiple layers. The natural silk cocoon containing all layers was cut as a rectangular shape as defined as total group. The inner and outermost layers were removed from the total group and the remained mat was defined as the middle group. The objectives of this study was to compare the total group with the middle group as a barrier membrane for the guided bone regeneration.

**Methods:**

The effects of these materials on the cellular proliferation and alkaline phosphatase (ALP) expression of MG63 cells were explored. For comparing bone regeneration ability, bilateral bone defects were created in calvarial areas in ten adult New Zealand white rabbits. The defects were covered with silk membranes of the middle group, with silk membrane of the total group used as the control on the contralateral side. The defects were allowed to heal for 4 and 8 weeks. Micro-computerized tomography (μCT) and histological examination were performed.

**Results:**

The middle group exhibited a higher MTT value 48 and 72 h after treatment compared to the total group. ALP expression was also higher in the middle group. The results of μCT and histologic examination showed that new bone formation was significantly higher in the middle group compared to the total group 8 weeks postoperatively (*P* < 0.05).

**Conclusions:**

In conclusion, the middle layer of the silk cocoon supports guided bone regeneration better than unprocessed silk cocoon.

## Background

Sufficient alveolar bone volume is a prerequisite for dental implant treatment for edentulous patients, assisted by direct bone regeneration. Guided bone regeneration (GBR) is a technique that compensates for the lost alveolar bone, in which a membrane acts as a barrier to block epithelial migration [1]. The idea of a barrier membrane dates back to Bassett et al., who designed cellulose acetate filters to support the regeneration of nerves and tendons [2]. The concept of guided bone regeneration originated with attempts to gain space for the regeneration of osseous defects via the utilization of the barrier membrane [3]. Ideal GBR membranes require adequate mechanical stability in addition to optimal biological properties [4].

A variety of membranes are commercially available for use in the clinic, including bioresorbable and non-resorbable membranes, with collagen membranes and titanium-reinforced polytetrafluoroethylene (Gore-Tex®) as typical examples of these two types, respectively. Bioresorbable collagen membranes, mainly prepared from porcine collagen [5], are mechanically weak and are unable to maintain space [6], whereas the non-resorbable Gore-Tex® has sufficient mechanical strength to support space even for vertical bone augmentation [7]. Despite this advantage of providing support to the osseous defect, the effectiveness of Gore-Tex® is limited due to the risk of membrane exposure leading to bone graft failure [4]. For the manufacturing collagen membranes, cross-linking agents, such as diphenylphosphorylazide or glutaraldehyde, have been used [8, 9]. Cross-linking agents can be cytotoxic during the healing phase [10]. With respect to the mechanical properties, Gore-Tex® membrane is easily torn in wet conditions and difficult to adapt and suture [11].

The aim of this study was to overcome the shortcomings of the existing membranes by developing new barrier membrane from silk protein. Silk is a natural macromolecule originating from *Bombyx mori* that mainly consists of two proteins, fibroin and sericin [12–15]. Fibroin is the structural center of silk, with a specific fibroin layer giving the silk tensile strength. The high glycine and alanine content in silk fibroin leads to tightly packed beta membranes, resulting in rigidity and tensile strength [15]. Silk fibroin can increase alkaline phosphatase activity and type I collagen expression in MG63 cells [16]. As both proteins are bone formation marker, silk fibroin has been widely used as a scaffold for bone graft [12]. The first silk barrier membrane was produced by electrospinning, which requires a large facility for mass production [17]. However, silk fibroin film was found to be fragile and dissolved easily in aqueous solution when it was used a barrier membrane [18].

Sericin is the gum coating the surface of fibroin, allowing fibroin molecules to stick to each other. Sericin is under investigation as a wound dressing material [19], and silk sericin-capped silver nanoparticles exhibited antibacterial activity against many gram-positive bacteria and gram-negative bacteria. Recent reports on the non-toxic nature of sericin [20] supported the use of whole silkworm-cocoon-derived silk membranes [21]. Because of an early report that sericin evoked IgE and IgG antibody reactions in humans [22], silk fibroin was purified from sericin by boiling silk cocoons in an alkaline solution (degumming) to remove the source of the immunologic reaction. In this study, we deliberately omitted the degumming process to avoid removing sericin. Silk from the cocoons of silkworms are known to have extraordinary mechanical properties [23], and the mechanical peeling of the silk cocoon generates membranes of different thicknesses [24], each of which could be utilized as barrier membranes. Our previous studies compared silkworm-cocoon-derived silk membranes with existing barrier membranes [25] and studied the effect of thickness [21]. Silkworm-cocoon-derived silk membranes showed comparable ability of bone regeneration compared to collagen and Gore-Tex® membrane [25]. Thicker silkworm-cocoon-derived silk membranes had better bone formation than the thinner one [21]. However, these studies used only the middle portion of the silkworm cocoon. Therefore, the process for removing the inner and the outermost layers of the silkworm cocoon is still needed. However, the reason of removing the inner and the outermost portions is unclear. At first, they were removed because of contamination from the moth pupas or the outer environment. This may be cleaned by several methods without removing the layers. If the natural cocoon has similar bone formation ability to that of the middle portion, removing the inner and the outermost layers of silkworm cocoon will not be needed. This will simplify the production process and reduce the cost for the production.

Silk cocoon is composed of multiple layers. After removing the inner and outermost layers, the remained membrane was assigned as the middle group. The objective of this study was to compare natural silk cocoons with the membranes of the middle group as barrier membranes for guided bone regeneration. The membranes were examined by scanning electron microscopy and osteoblast-like cell culture. The silk membranes were grafted into the calvarial bone defects of rabbits, and the new bone formation of each group was compared by micro-computerized tomography (μCT) and histological examination.

## Methods

### Silk membrane

**Fig. 1:**
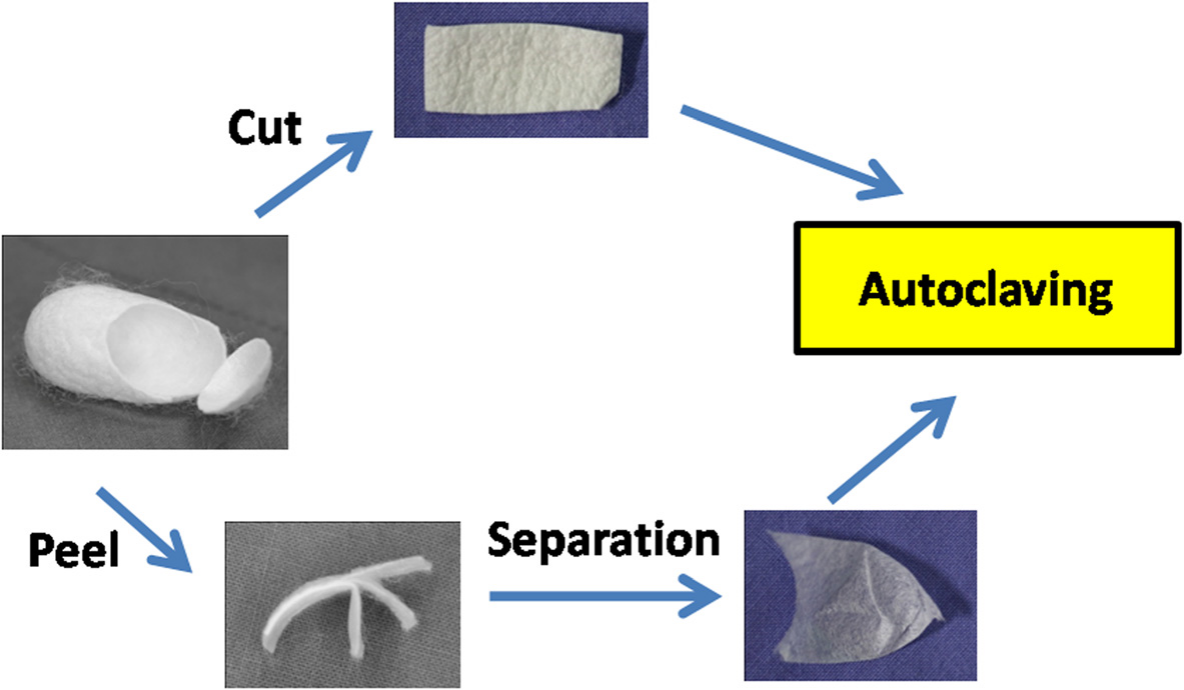
Schematic drawings for the silk membrane fabrication

*B. mori* cocoons were obtained from the Rural Development Administration (Jeonju, Korea). The raw cocoons were cut to 1 cm × 1-cm size pieces and used for the total group. The inner and outermost layers of the cocoons were removed by mechanical peeling (Fig. 1). The thickness of the removed portion was approximately 0.2 mm for each. Then, the remaining middle layer was cut as 1 cm × 1 cm and used for the middle group. Each membrane was then washed with ethanol. Subsequently, the silk membranes were sterilized by autoclave at 121 °C and 1.5 atmospheric pressure for 30 min. The thickness was measured by caliper.

### SEM imaging

All materials were prepared for scanning electron microscope (SEM) examination. The prepared specimens underwent SEM analysis at Gangneung Center in Korea Basic Science Institute (KBSI). After immobilization of the samples on the plate, each sample was coated with gold. Specimens were imaged using a SU-70 microscope (Hitachi, Japan) operating at 5 keV.

### Cell culture

In vitro tests were performed using MG-63 osteoblast-like cells (ATCC, Manassas, VA, USA). The cells were grown to 80 % confluence in Dulbecco’s modified Eagle’s medium-high glucose (PAA Laboratories, Linz, Austria) containing 1 % penicillin/streptomycin (100×), supplemented with 10 % fetal bovine serum (PAA Laboratories, Etobicoke, ON, Canada) at 37 °C in an atmosphere of 5 % CO_2_ and 99 % relative humidity. Media were changed every 3 days.

The mat from the total group and middle group was cut as 1 cm^2^-sized rectangular shape. The samples were sterilized by autoclave at 121 °C and 1.5 atmospheric pressure for 30 min and passivated in DMEM culture medium for 12 h, prior to seeding cells. A seeding density of 20,000 cells/cm^2^ was used, and the specified amounts of cells were seeded using 1 ml of DMEM medium. The plates were incubated in a humidified environment for a period of up to 14 days, and the medium was changed every second day. At specific time intervals, samples were analyzed for tetrazolium salt 3-(4, 5-dimethylthiazole-2-yl)-2,5-diphenyltetrazolium bromide (MTT) assay and alkaline phosphatase (ALP) assay.

### MTT assay and alkaline phosphatase assay

Cell viability was quantified after 24, 48, and 96 h of culture growth using the tetrazolium salt MTT assay. Briefly, cells were incubated with MTT solution (Cell proliferation kit I; Roche Molecular Biochemicals, Mannheim, Germany) in six-well plates for 4 h at 37 °C in an atmosphere of 5 % CO_2_ and 99 % relative humidity. Formazan crystals were solubilized with DMSO at room temperature overnight, and the product concentration was determined by measuring the absorbance at 540 nm with a Victor Multilabel counter (Perkin-Elmer-Wallac, Freiburg, Germany).

ALP was evaluated by the transformation of p-nitrophenylphosphate into p-nitrophenol at 37 °C and pH 10.2 using appropriate reactive chemicals (all from Sigma, St. Louis, MO, USA), while the specific activity of ALP was calculated based on the protein concentration of lysates determined using a commercially available colorimetric assay (#71230, AnaSpec, Freemont, CA, USA). The product concentration was determined by measuring the absorbance at 415 nm with an iMark microplate absorbance reader (BioRad, Hercules, CA, USA). ALP standard was prepared in a serial dilution of 1:50 and detected using an absorbance of 405 nm.

### Animals and surgical procedure

Ten 12-week-old New Zealand white rabbits with an average weight of 2.3 kg (range 2.0–2.5 kg) were used for this experiment. This experiment was approved by the Institutional Animal Care and Use Committee of the Gangneung-Wonju National University, Gangneung, Korea (GWNU-2014-5).

General anesthesia was administered by intramuscular injection of a combination of Zoletil 50 (15 mg/kg; Vibac, Carros, France) and Rumpun (0.2 mL/kg; Bayer Korea, Seoul, Korea). The cranial area was shaved and disinfected with povidone-iodine. Two percent lidocaine with epinephrine (1:100,000) was applied to the cranial area. A midline incision was made in the sagittal plane of the skull, and subperiosteal dissection was performed to expose the calvaria. A trephine bur was used under saline irrigation to create a calvarial defect. Two 8-mm-diameter defects were created, one on each side of the midline. The calvarial defects were covered with the total or middle membranes.

Following treatment, the pericranium and skin were closed in layers with 3–0 black silk (AILEE, Busan, Korea). After surgery, the rabbits received 1 mg/kg gentamicin (Kookje, Seoul, Korea) and 0.5 mL/kg pyrin (Green Cross Veterinary Products, Seoul, Korea) intramuscularly, three times daily for 3 days.

Each rabbit was individually caged and received food and water. All animals were killed at 4 or 8 weeks after surgery. Specimens were separated and fixed in 10 % formalin. After μCT analysis, histological analysis was performed.

### μCT analysis

The prepared specimens were analyzed by μCT using an animal PET/CT/SPECT system (Inveon, Siemens, Erlangen, Germany) at the Ochang Center of the Korea Basic Science Institute. The μCT scanner was set to 80 kV for the X-ray tube, with a 500-μA current for the X-ray source and a 210-ms exposure time. The detector and X-ray source were rotated through 360° in 360 steps, with 30 calibration exposures. The system magnification was set to produce an axial field of view (FOV) of 30.74 mm and a trans-axial FOV of 30.74 mm. The scanned images were reconstructed using the Inveon Research Workplace software (Siemens). Gross profiles of the specimens were obtained from reconstructed three-dimensional images. Because the initial defect was round in shape with an 8.0-mm diameter, the region of interest (ROI) was set based on the initial defect size and shape. The ROI of each specimen was analyzed for bone volume (BV).

### Histomorphometric evaluation

The calvarial samples were harvested, decalcified in 5 % nitric acid for 5 days, and dehydrated in ethyl alcohol and xylene. After separation of the parietal bones through the midline sagittal suture, the calvarial samples were embedded in paraffin blocks. The paraffin blocks were sliced into sections that were then stained with hematoxylin and eosin. The section with the largest defect area was selected, along with sections 50 μm proximal and distal to the largest defect section.

The staining procedure for hematoxylin and eosin staining was as follows. First, de-wax and hydrate paraffin sections. The slide was stained in hematoxylin for 5 min. Overstained sections can easily be differentiated by agitating for a second in acid-alcohol, then washing in tap water for 5 min. The slides were immersed in eosin for 30 s and then wash them in running tap water for 1 min. The slides were dehydrated and clear in xylene.

Digital images of the selected sections were captured with a digital camera (DP-73; Olympus, Tokyo, Japan). The images were analyzed by Sigma Scan pro (SPSS, Chicago, IL). The new bone formation was calculated as the percentage of newly formed bone in the calvarial defect area.

### Statistical analysis

SPSS for window ver. 19 (IBM Co., Armonk, NY, USA) were used for statistical analysis. The differences between the mean values of the total group and the middle group in the MTT and alkaline phosphatase (ALP) assays were evaluated by independent sample *t* tests. Paired *t* test was used for comparison the samples within the same animal. The level of significance was set as *P* < 0.05.

## Results

### Gross appearance

**Fig. 2:**
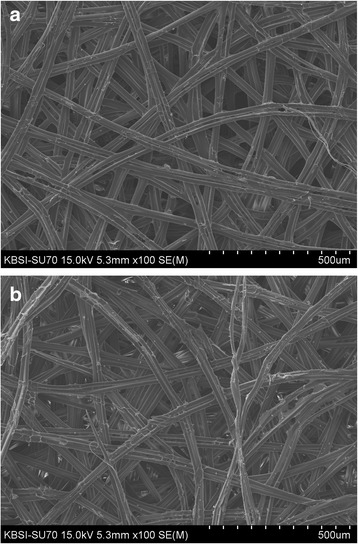
SEM images of the silk membrane. **a** Unprocessed control. **b** Middle layer group

The thickness of the total group was 0.61 ± 0.02 mm and that of the middle group was 0.32 ± 0.03 mm. Gross morphology was observed by SEM. No striking differences in morphology were observed between groups (Fig. 2).

### MTT assay and ALP assay

**Fig. 3:**
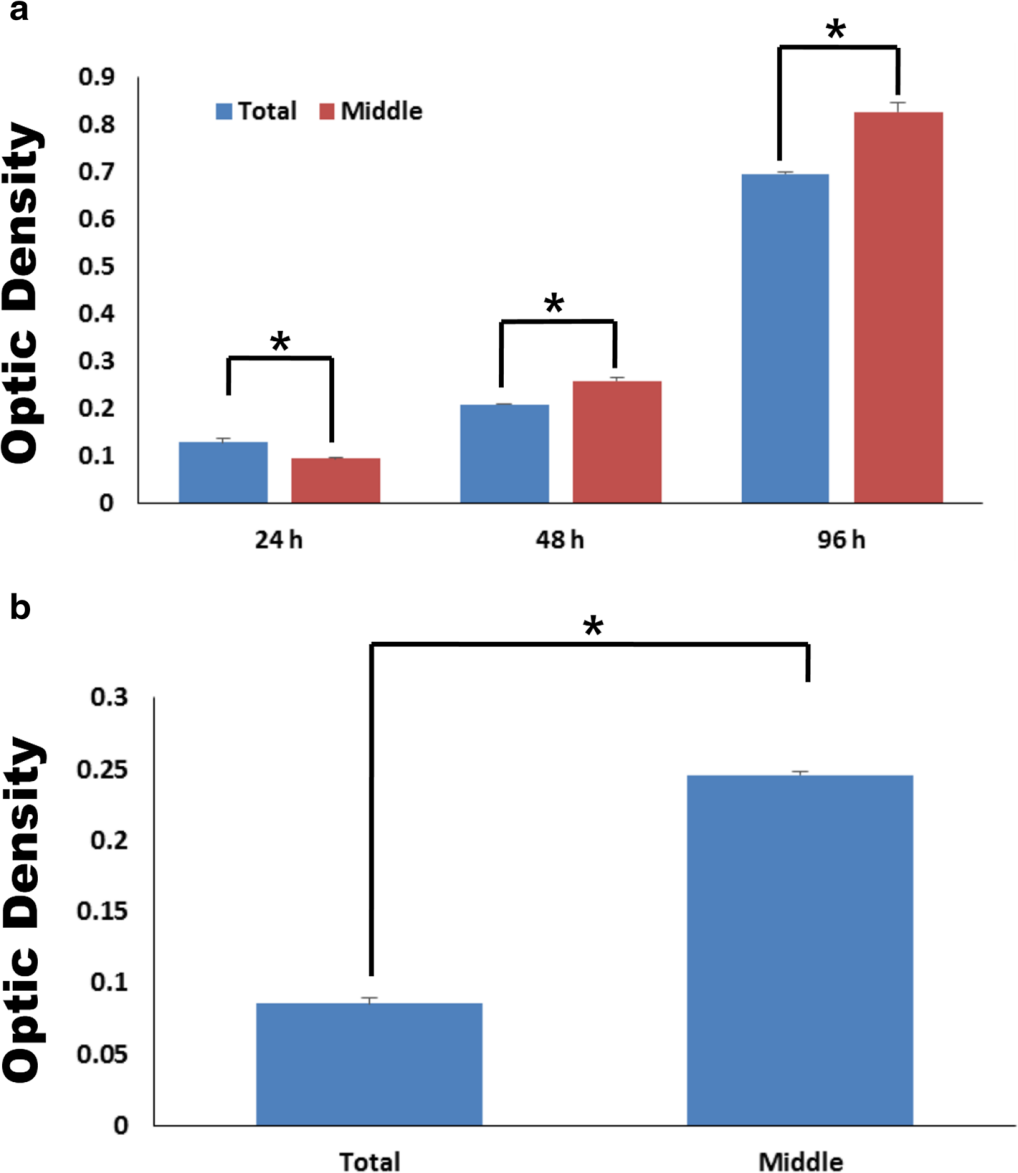
The biological evaluations. **a** MTT assay of MG63s on unprocessed silk cocoon (total group) and middle layer silk cocoon (middle group) membranes (after 24, 48, 72 h of cell culture, *asterisk* significantly different at *P* < 0.05) and **b** alkaline phosphatase assay of MG63s after 72 h of cell culture (*asterisk* significantly different at *P* < 0.05). *Error bars* mean standard deviation

Compared with the total group, MG-63 cells treated with the middle layer exhibited decreases in MTT activity at 24 h after application (*P* < 0.001). MG-63 cells treated with the middle layer exhibited increases in MTT activity at 48 and 96 h after appliscation (*P* < 0.001) (Fig. 3a).

The relative ALP activity of the middle group was approximately threefold higher compared with that of the total group. The difference between groups was statistically significant (*P* < 0.001) (Fig. 3b).

### In vivo test

**Fig. 4:**
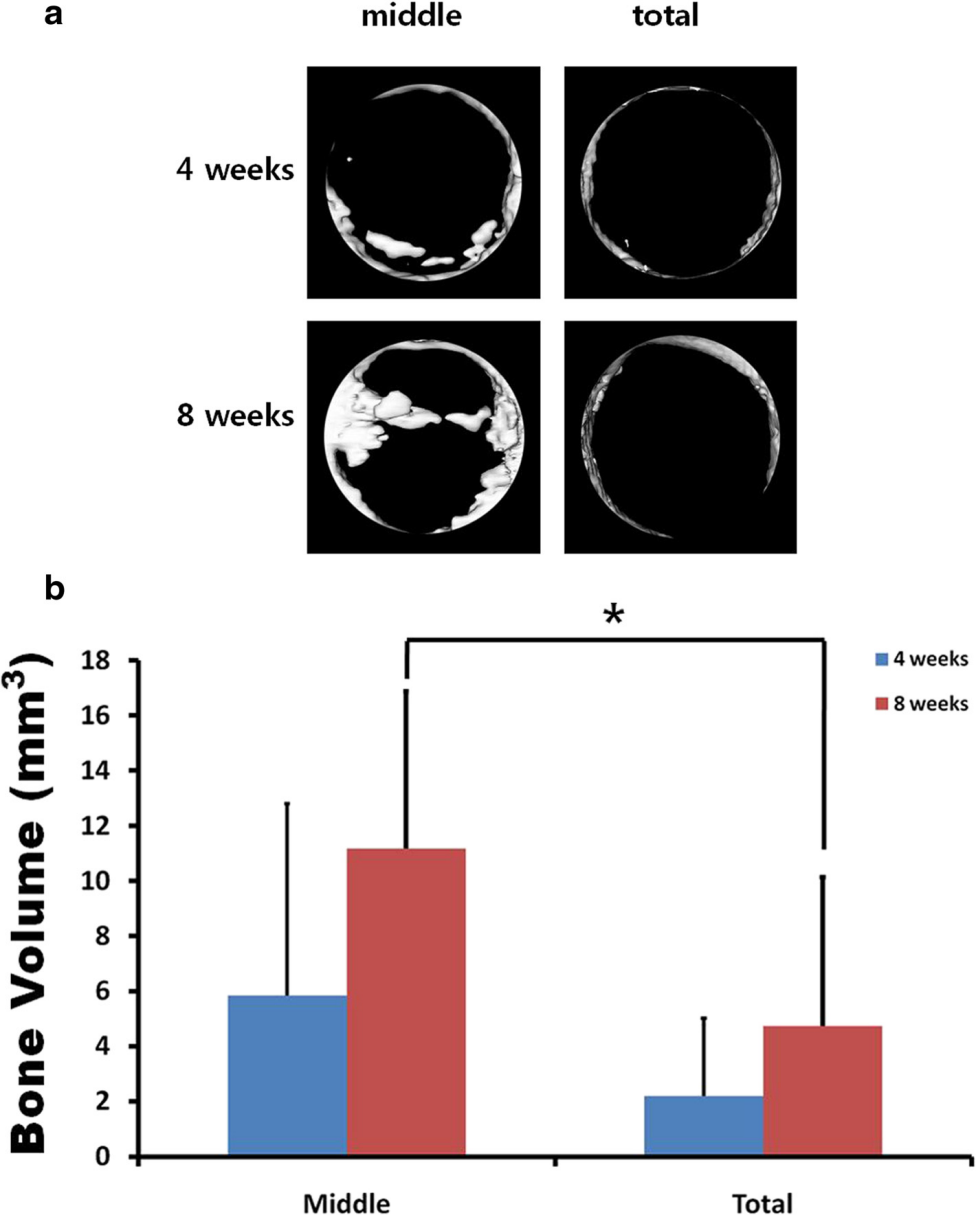
Radiographic examination. **a** Micro-computed tomography. **b** The bone volume analysis (*asterisk* significantly different at *P* < 0.05)

The results of the μCT analysis are presented in Fig. 4a. The average values of the two measured variables were higher in the middle membrane group than those in the total membrane group at both 4 and 8 weeks. The bone volume in the middle group at 4 weeks after the operation was 5.85 ± 6.94 mm^3^, and the volume in the total group was 2.20 ± 2.81 mm^3^. This difference was not statistically significant (*P* > 0.05). The bone volume in the middle group at 8 weeks after the operation was 11.17 ± 5.69 mm^3^ and that in the total group was 4.73 ± 5.38 mm^3^. This difference was statistically significant (*P* = 0.048) (Fig. 4b).

**Fig. 5:**
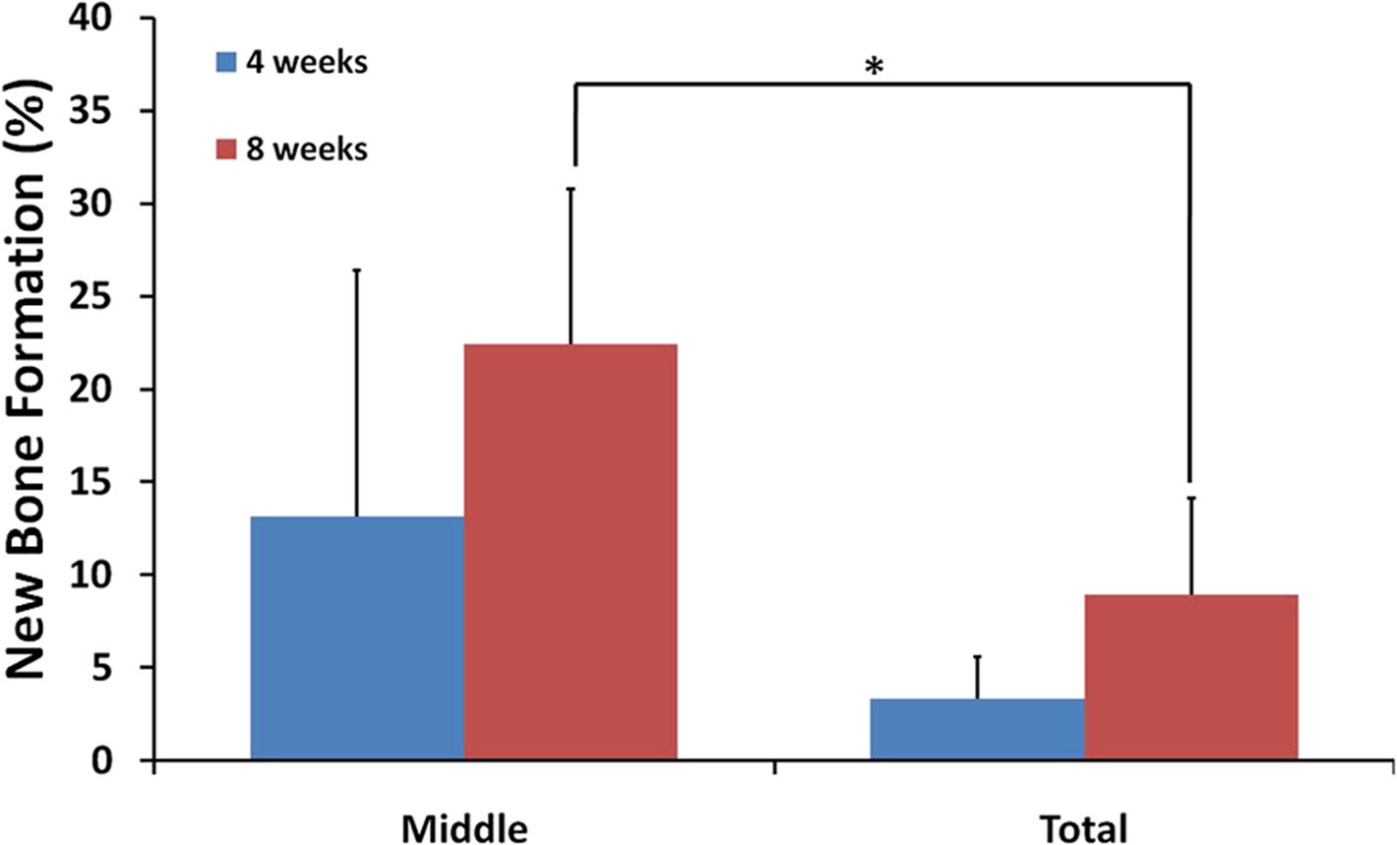
Histological analysis of new bone formation (*asterisk* significantly different at *P* < 0.05, *n* = 5)

The results of the histological analysis are presented in Fig. 5. The average values of the two measured variables were higher in the middle membrane group than those in the total membrane group at both 4 and 8 weeks. The new bone formation in the middle group at 4 weeks after the operation was 13.14 ± 13.23 % and that in the total group was 3.32 ± 2.23 %. This difference was not statistically significant (*P* > 0.05). The new bone formation in the middle group at 8 weeks after the operation was 22.41 ± 8.38 % and that in the total group was 8.94 ± 5.16 %. This difference was statistically significant (*P* = 0.016).

**Fig. 6:**
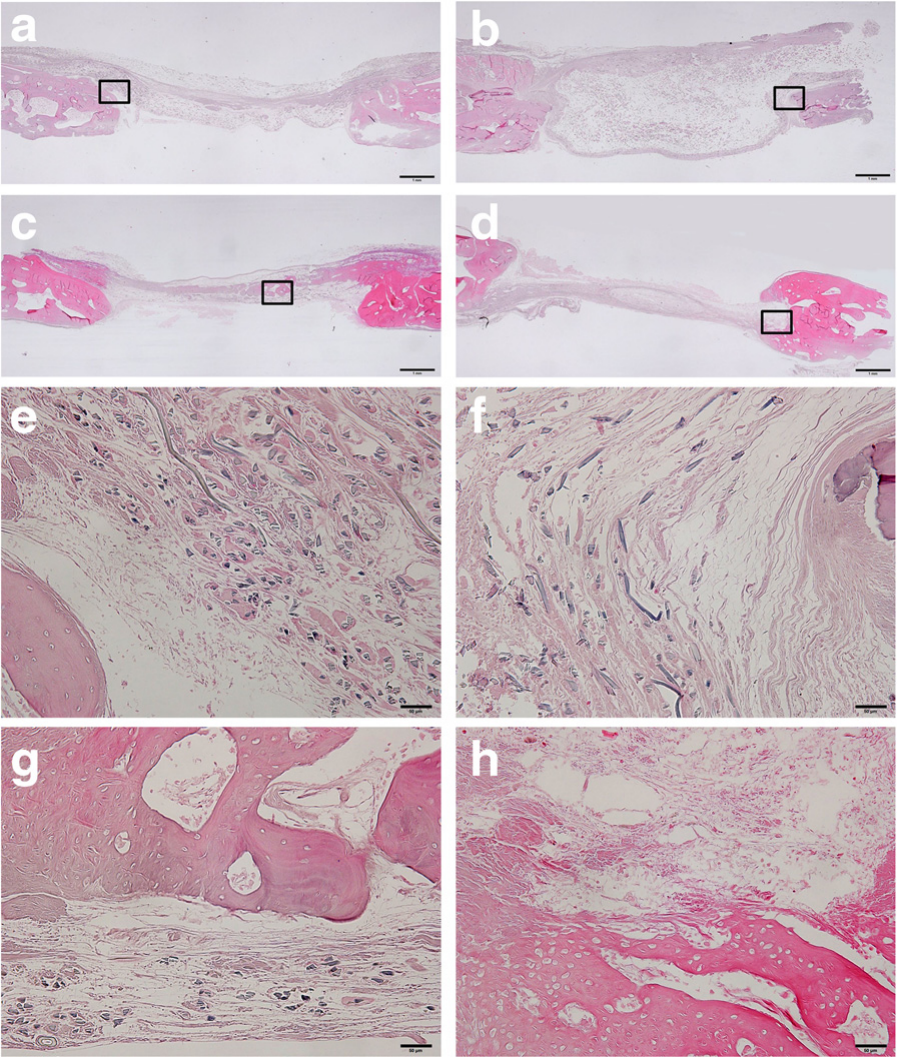
The histological examination. The residual membrane at 4 weeks after operation was less observed in the middle group (**a**) than in the total group (**b**) (bar = 1.0 mm, hematoxylin and eosin stain). New bone formation at 8 weeks after operation was more observed in the middle group (**c**) than in the total group (**d**) (bar = 1.0 mm, hematoxylin and eosin stain). The boxed area of low magnification views (**a**–**d**) were magnified and shown in **e**–**h**, respectively (bar = 50 μm)

Less residual membrane was observed in the specimen at 8 weeks after the operation compared to the specimen at 4 weeks after the operation in both groups (Fig. 6a, b). No foreign body giant cells were observed in either group (Fig. 6e, f). Interestingly, new bone formation was observed above the membrane in the middle group 8 weeks after the operation (Fig. 6c, g). In the total group, new bone formation was observed on both sides of the membrane (Fig. 6d, h).

## Discussion

This study found that the middle layer of silk cocoon induced better new bone formation than unprocessed silk cocoon. As the process of separating the middle layer did not involve any chemical treatment, the gross morphology observed by SEM was similar between the two groups (Fig. 2). As the membrane from the middle layer of the cocoon was thinner than the unprocessed silk cocoon, dentists may find it easier to handle the middle group membrane. To the best of our knowledge, no reports have compared the unprocessed silk cocoon and the middle layer of silk cocoon in the GBR technique.

MG-63 cells treated with the middle layer of the cocoon of *B. mori* showed decreased MTT activity 24 h after treatment, but the activities were increased at 48 and 96 h (Fig. 3a, *P* < 0.001). These results for the middle group were statistically significant compared to those of the total group, which shows that the overall cellular toxicity of the middle group is favorable. The relative ALP activity of the middle group was dramatically higher than that of the total group, and this result was statistically significant (Fig. 3b, *P* < 0.001). Taking these results of the cellular studies into consideration, the silk membrane made of the middle layer of the cocoon of *B. mori* exhibits superior safety and efficacy to the membrane prepared from total cocoon.

The results of the animal study were similar to those of the cellular experiments. The results of the μCT analysis showed that the bone volume in the middle group was significantly higher than that in the total group at 8 weeks postoperatively (Fig. 4, *P* = 0.048). The histological analysis demonstrated significantly greater new bone formation at 8 weeks after the operation in the middle group compared with the total group (Fig. 5, *P* = 0.016). The inner layer of silk cocoon has a lower porosity and a smaller average diameter of silk than the outer layer [24]. Each layer of the silk cocoon has a different silk fibroin to sericin ratio [26]. The outermost layer has over 30 % of sericin, and the inner most layer has less than 20 % of sericin [26]. Besides silk fibroin and sericin, other natural impurities, such as waxes for protecting the moth pupas are also found [26]. The anti-inflammatory effects of sericin [27] may also induce immune reactions upon physical association with fibroin-silk fibers [28]. The graft composition determines whether sericin exerted a positive or negative effect on graft success. The middle layer induced the best new bone formation among divided silk cocoon layers (Fig. 6g). The middle layer may have the optimal composition ratio of silk fibroin and sericin for bone formation. Comparing between different thicknesses of the same silk cocoon middle layer, the thicker membrane leads to better new bone formation [21]. Therefore, the silk mat from the middle layer only seemed to be proper for GBR technique. The inner and outermost layers of the silk cocoon should be removed before use for the GBR technique. Except for the different amino acid composition among layers, other differences among layers are still unclear. The exact mechanism should be clarified by further studies.

Less residual membrane was observed in the specimen 8 weeks after operation compared to the specimen 4 weeks after operation in both groups (Fig. 6). As the membranes derived from silk cocoon are composed of amino acid, they may underwent proteolysis gradually. Other membranes based on natural materials, such as collagen, are also biodegradable [17]. In case of collagen membrane, if it is not exposed to the oral environment, it is intact in the graft site until 28 days after implantation [29]. No foreign body giant cells were observed in either group. Interestingly, most previous silk fibroin grafts have elicited many foreign body giant cells. Sericin exerts an anti-inflammatory effect [27]. Sericin or other components of the silk cocoon may inhibit foreign body giant cell formation. In this study, both membranes had been sterilized by autoclave, and their mechanical properties after autoclaving were proved to be intact by the measured stress-strain curve, indicating that the membranes were not damaged by autoclaving (data not shown). Therefore, the silk cocoon-derived membrane can be used for clinical trials after sterilization.

## Conclusions

In conclusion, the middle layer of the silk cocoon was more appropriate for use in the GBR technique than the unprocessed silk cocoon. However, few data are available on the clinical applications of silk membranes. As the processing procedure used here is simple and natural, clinical trials should be carried out using materials prepared using this procedure.

## Abbreviations

ALP: alkaline phosphatase
GBR: guided bone regeneration
MTT: tetrazolium salt 3-(4, 5-dimethylthiazole-2-yl)-2,5-diphenyltetrazolium bromide
SEM: scanning electron microscope
μCT: micro-computerized tomography
